# Supplementary figures and images for: Natural variants of ELF3 affect thermomorphogenesis by transcriptionally modulating PIF4-dependent auxin response genes
Source: BMC Plant Biol. 2015 Aug 14;15:197. doi: 10.1186/s12870-015-0566-6 (PMC4535396; doi:10.1186/s12870-015-0566-6)

**A**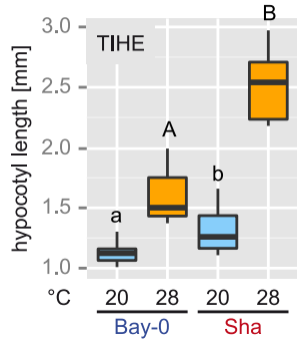**B**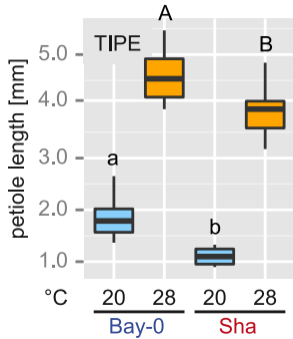**C**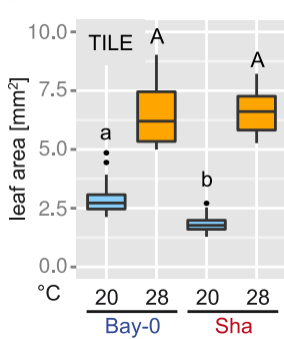

Supplement: Additional file 1: Figure S1. — Temperature-induced growth responses in Bay-0 and Sha. Absolute values for (A) temperature-induced hypocotyl elongation (TIHE), (B) temperature-induced petiole elongation (TIPE), and (C) temperature-induced leaf expansion (TILE). Data correspond to the relative data presented in Fig. 1. Box plots show medians (horizontal bar), interquartile ranges (IQR, boxes), and data ranges (whiskers). Black dots mark outliers (defined as > 1.5 × IQR). Statistical differences were assessed by one-way ANOVA and Tukey HSD (P < 0.05) and are denoted by different lower- and uppercase letters for 20 °C and 28 °C values, respectively. [file 12870_2015_566_MOESM1_ESM.pdf]

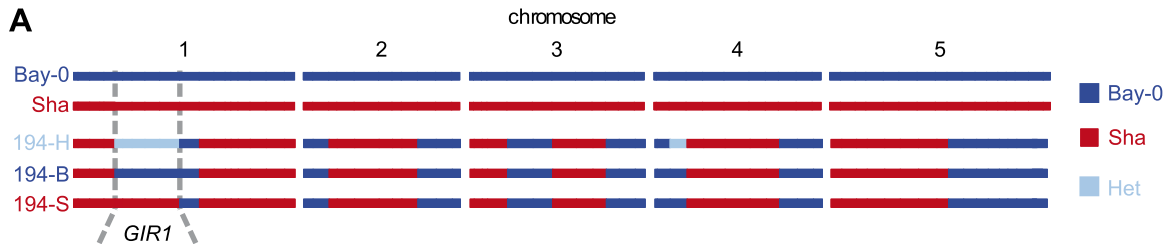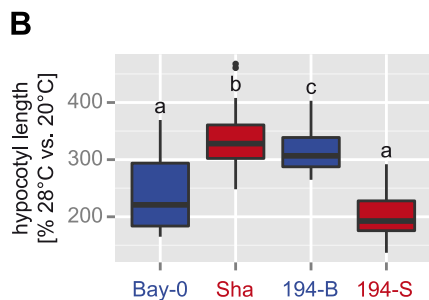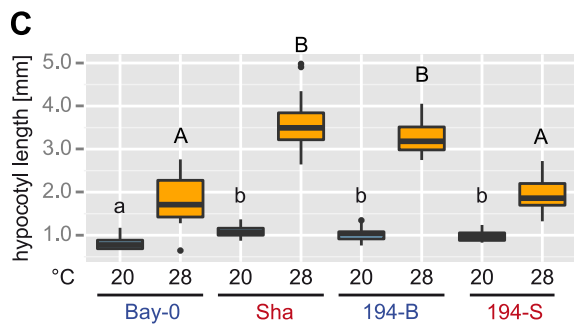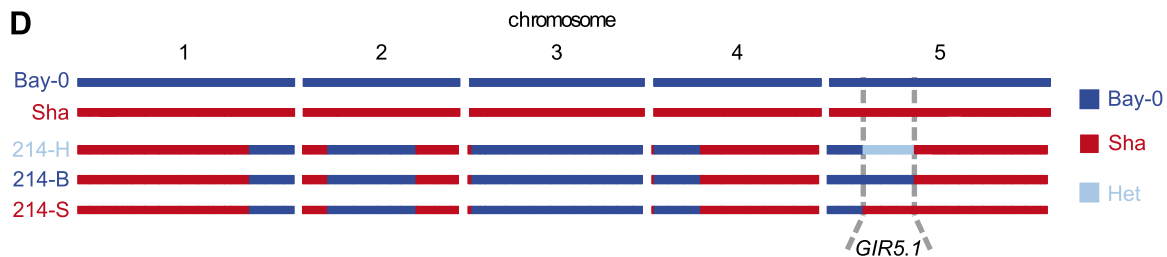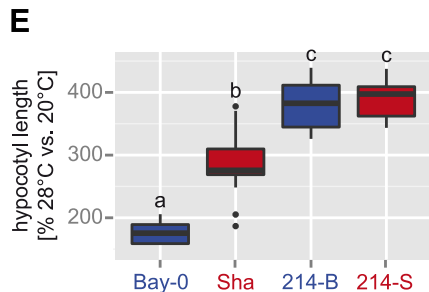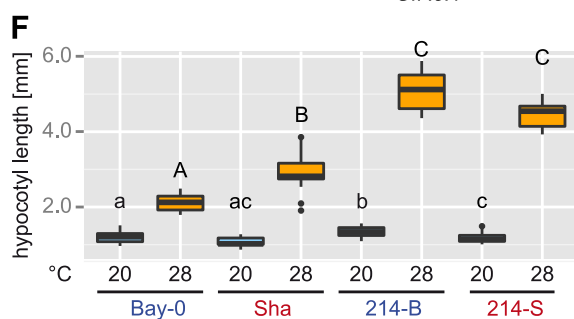

Supplement: Additional file 4: Figure S2. — Validation of the GIR1 QTL. (A) Haplotype overview of the heterogeneous inbred family (HIF) 194 that segregates for Bay-0 and Sha within the GIR1 interval and was used for validation and mapping of the GIR1 QTL. (B) Box plots show relative (28 °C/20 °C in %) hypocotyl length of 10 days-old seedlings derived from the absolute hypocotyl length data presented in (C). (D) Haplotype overview of the heterogenous inbred family (HIF) 214 that segregates for Bay-0 and Sha within the GIR5.1 interval and served for the attempted validation of this QTL. (E) Box plots show relative (28 °C/20 °C in %) hypocotyl length of 10 days-old seedlings derived from the absolute hypocotyl length data presented in (F). (B,C,E,F) Horizontal bars, boxes, and whiskers show medians, interquartile ranges (IQR), and data ranges, respectively. Black dots mark outliers (defined as > 1.5 × IQR). (B+E) Different letters denote statistical differences in temperature responses as assessed by two-way ANOVA (P < 0.05) of the absolute hypocotyl length data. (C+F) Statistical differences were assessed by one-way ANOVA and Tukey HSD (P < 0.05) and are denoted by different lower- and uppercase letters for 20 °C and 28 °C values, respectively. The significant differences in TIHE observed for the parental lines Bay-0 and Sha was not reflected by the two HIF lines 214-B and 214-S that carried a Bay-0 or Sha allele within the GIR5.1 interval, respectively. As such, the GIR5.1 QTL could not be validated with the available genetic material. [file 12870_2015_566_MOESM4_ESM.pdf]

**A**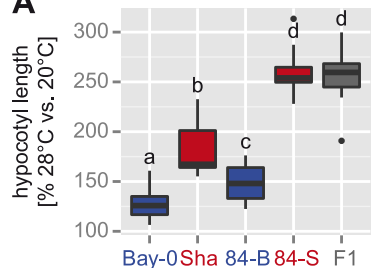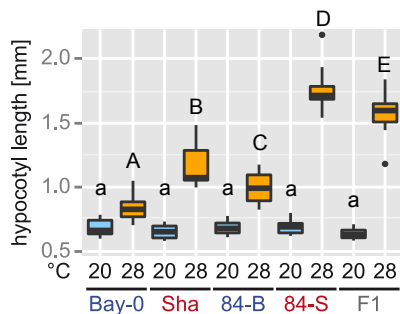**B**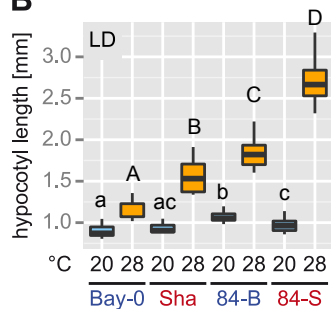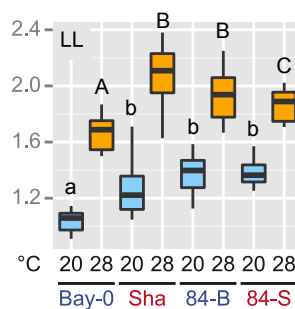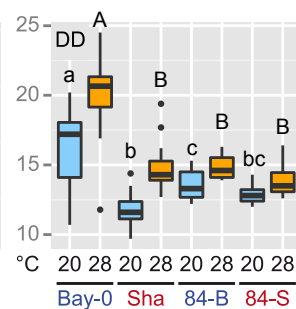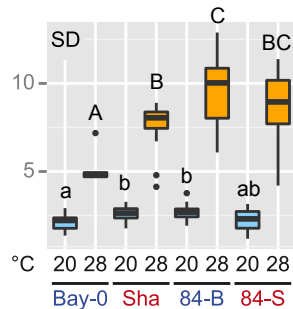**C**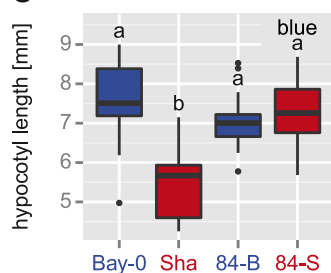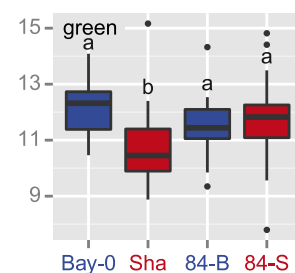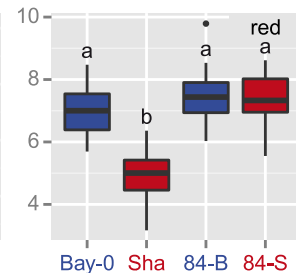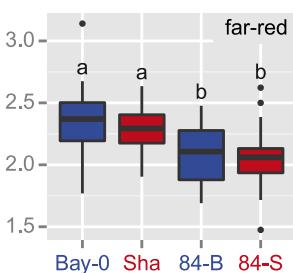

Supplement: Additional file 5: Figure S3 — Effect of altered light conditions on GIR2.1-mediated hypocotyl elongation. (A) Box plots show relative (28 °C/20 °C in %) and absolute hypocotyl length of 10 days-old seedlings of Bay-0, Sha, and HIF lines homozygous for either Bay-0 (84-B) or Sha (84-S) in the GIR2.1 interval. Horizontal bars, boxes, and whiskers show medians, interquartile ranges (IQR), and data ranges, respectively. Black dots mark outliers (defined as > 1.5 × IQR). F1 plants derived from a cross of 84-B and 84-S correspond to the haplotype 84-H in Fig. 2a and illustrate the dominance of the Sha over the Bay-0 allele. Different letters denote statistical differences in temperature responses as assessed by two-way ANOVA (i.e. genotype x treatment effect, P < 0.05) of the absolute hypocotyl length data. (B) Absolute hypocotyl length corresponding to the relative data presented in Fig. 2b. Statistical differences were assessed by one-way ANOVA and Tukey HSD (P < 0.05) and are denoted by different lower- and uppercase letters for 20 °C and 28 °C values, respectively. (C) Hypocotyl length in monochromatic light conditions. Significant differences among Bay-0 and Sha are observed in 4 days-old seedlings grown at 20 °C in constant blue (4.93 μmol m−2 sec−1), green (0.32 μmol m−2 sec−1), or red (0.89 μmol m−2 sec−1) light. These differences seem to be regulated independent of GIR2.1 as 84-B and 84-S did not differ in their growth response. No differences among genotypes were detected in seedlings grown in far-red (0.024 μmol m−2 sec−1) light. Statistical differences were assessed by one-way ANOVA and Tukey HSD (P < 0.05) and are denoted by different lower- and uppercase letters for 20 °C and 28 °C values, respectively. [file 12870_2015_566_MOESM5_ESM.pdf]

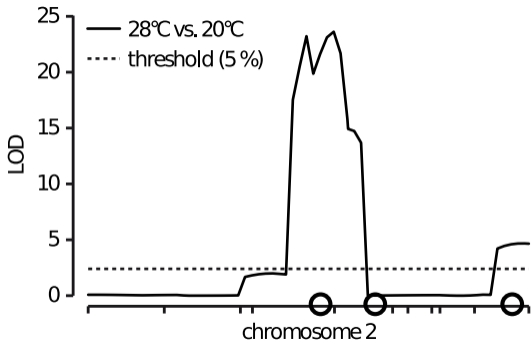

Supplement: Additional file 6: Figure S4. — GIR2 constitutes a ghost QTL. Setting additional covariates in the GIR2.1 target region separates the single peak into two linked peaks (compare with Fig. 1d), indicating the potential existence of two linked loci. Tick marks on the x axis correspond to molecular markers in the genetic map of the Bay-0 and Sha mapping population. Circles on the x axis show co-variates set for composite interval mapping. [file 12870_2015_566_MOESM6_ESM.pdf]

**A**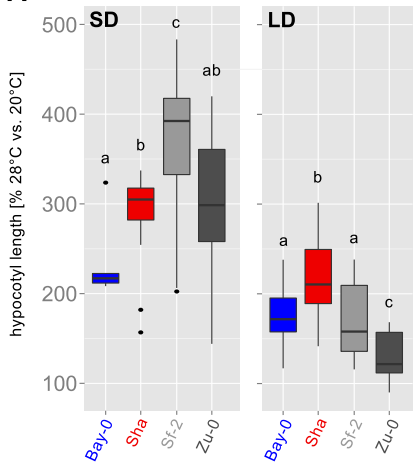**B**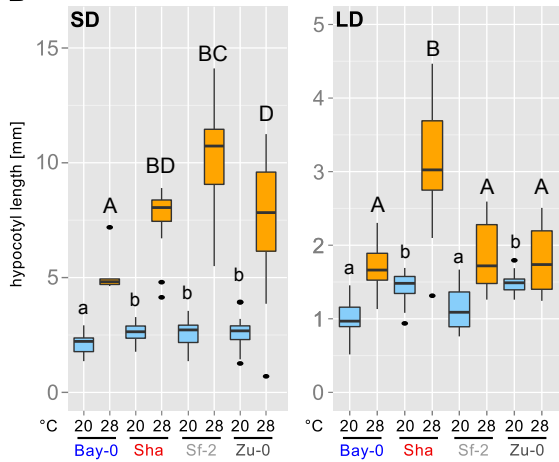

Supplement: Additional file 7: Figure S5. — Photoperiod and allele effects on ELF3-mediated TIHE. TIHE comparison of 7 days-old seedlings grown either in short day (SD) or long day (LD) photoperiods. Box plots show (A) relative (28/20 °C in %) and (B) absolute hypocotyl length for Bay-0, Sha and MAGIC population parental lines Sf-2 and Zu-0 that also carry polymorphisms in ELF3. While Sf-2 and Zu-0 show a strong TIHE response in SD, the response for Sf-2 and Zu-0 is much weaker under LD. Horizontal bars, boxes, and whiskers show medians, interquartile ranges (IQR), and data ranges, respectively. Black dots mark outliers (defined as > 1.5 × IQR). (A) Different letters in denote statistical differences in temperature responses as assessed by two-way ANOVA (i.e. genotype x treatment effect, P < 0.05) of the absolute hypocotyl length data. (B) Statistical differences were assessed by one-way ANOVA and Tukey HSD (P < 0.05) and are denoted by different lower- and uppercase letters for 20 °C and 28 °C values, respectively. [file 12870_2015_566_MOESM7_ESM.pdf]
