# Supplementary material for: Natural variants of ELF3 affect thermomorphogenesis by transcriptionally modulating PIF4-dependent auxin response genes
Source: BMC Plant Biol. 2015 Aug 14;15:197. doi: 10.1186/s12870-015-0566-6 (PMC4535396; doi:10.1186/s12870-015-0566-6)
Supplement: Additional file 2: Table S1. — Descriptive statistics of phenotypes analyzed in the Bay-0 × Sha population. [file 12870_2015_566_MOESM2_ESM.pdf]

trait	20 °C	28 °C	TIHE[e]
**n[a]**	400 (4 | 16)	395 (3 | 22)	387 (4 | 22+7)
**mean ± SD[b]**	1.138 ± 0.173	2.033 ± 0.563	80.97 ± 51.86
**range (min–max)[b]**	0.711 – 1.680	0.976 – 3.781	-20.34 – 258.68
**SW-Test[c]**	0.0050	0.0000	0.0000
**skewness[b]**	0.376	0.949	0.906
**kurtosis[b]**	0.076	0.561	0.551
**BSH ± SD[d]**	0.6861	0.8266	0.898 ± 0.004

[A] Number of RILs included in the QTL analysis. Numbers in brackets indicate lines omitted from the analysis due to z-score threshold outside of 3 or experimental reasons (e.g. failure to germinate), respectively.

[b] Values determined after removing lines outside z-score threshold of three.

[c] Shapiro-Wilk-Test calculated after removing lines outside z-score threshold of three.

[d] Broad-sense heritability calculated only for lines present in all datasets (n=387).

[e] Temperature-induced hypocotyl elongation (28 °C vs. 20 °C).
