# Supplementary material for: Natural variants of ELF3 affect thermomorphogenesis by transcriptionally modulating PIF4-dependent auxin response genes
Source: BMC Plant Biol. 2015 Aug 14;15:197. doi: 10.1186/s12870-015-0566-6 (PMC4535396; doi:10.1186/s12870-015-0566-6)
Supplement: Additional file 3: Table S2. — QTL summary statistics. [file 12870_2015_566_MOESM3_ESM.pdf]

trait	cim QTL					stepwise QTL										
	QTL	Chr	position	LOD score	P-value	QTL	total R^2 (%)	Chr	position (cM)	closest marker	LOD score	95% CI (cM)	R^2 (%)	model	additive effect	F-test (P-value)
20℃	***GIR20_1***	1	2	5.43	0	***GIR20_1***	19.718	1	7	F21 M12	8.6	3-11	8.3487	y~**Q1**+Q2+Q3+Q1:Q3	0.0388 (0.0084)	$3.27*10^{-9}$
						GIR20_2		2	60	MSAT 2.10	2.7	24-64.6	2.5322	y~Q1+**Q2**+Q3+Q1:Q3	0.0302 (0.0086)	0.0005
	GIR20_3	3	15	7.85	0	***GIR20_3***		3	15	MSAT 305754	11.8	4-18	11.6479	y~Q1+Q2+**Q3**+Q1:Q3	-0.0598 (0.0094)	$2.4*10^{-12}$
28℃	***GIR28_1***	1	18	13.54	0	***GIR28_1***	52.603	1	18	IND 6375	24.0	17-20	15.3340	y~**Q1**+Q2+Q3+Q4+Q5+Q6+Q1:Q2	0.2314 (0.0209)	0
	GIR28_2	2	36	20.38	0	***GIR28_2***		2	36	MSAT 2.41	33.9	35-37	22.9690	y~Q1+**Q2**+Q3+Q4+Q5+Q6+Q1:Q2	-0.2833 (0.0210)	0
						GIR28_4		4	24.2	MSAT 4.35	2.8	13-30	1.5963	y~Q1+Q2+**Q3**+Q4+Q5+Q6+Q1:Q2	0.0718 (0.0199)	0.0003
	GIR28_5	5	36	8.05	0	***GIR28_5.1***		5	1	MSAT 500027	3.8	0-22	2.1614	y~Q1+Q2+Q3+**Q4**+Q5+Q6+Q1:Q2	-0.0885 (0.0211)	$3.3*10^{-5}$
						GIR28_5.2		5	43	MSAT 512110	8.0	36-47	4.6193	y~Q1+Q2+Q3+Q4+**Q5**+Q6+Q1:Q2	-0.1318 (0.0215)	$2.03*10^{-9}$
						GIR28_5.3		5	71.6	MSAT 5.12	4.8	69-88	2.7520	y~Q1+Q2+Q3+Q4+Q5+**Q6**+Q1:Q2		$3*10^{-6}$
TIHE	***GIR1***	1	25	5.98	0	***GIR1***	43.293	1	26	MSAT 108193	10.5	16-28	7.5838	y~**Q1**+Q2+Q3+Q4+Q5+Q2:Q4	14.8284 (2.0800)	$5.13*10^{-12}$
	GIR2	2	36	23.49	0	***GIR2.1***		2	35	MSAT 2.41	23.2	35-36	18.0554	y~Q1+**Q2**+Q3+Q4+Q5+Q2:Q4	-22.1834 (2.1771)	0
						GIR2.2		2	62	MSAT 2.22	5.2	59-64.6	3.6010	y~Q1+Q2+**Q3**+Q4+Q5+Q2:Q4	-11.5189 (2.3449)	$1.34*10^{-6}$
	GIR5	5	21	5.77	0	***GIR5.1***		5	35	NGA 139	7.2	29-40	5.0786	y~Q1+Q2+Q3+**Q4**+Q5+Q2:Q4	-12.8525 (2.4091)	$8.36*10^{-8}$
						GIR5.2		5	71	MSAT 5.12	2.6	56-91.2	1.7793	y~Q1+Q2+Q3+Q4+**Q5**+Q2:Q4	-7.4189 (2.1485)	0.0006

Chr, chromosome; **CI**, confidence interval; **cim,** composite interval mapping**; cM**, centimorgan; ***GIR**, GIRAFFE*; **LOD**, logarithm of odds; **QTL**, quantitative trait locus; $\mathbf{R^2}$, phenotypic variance; **TIHE**, temperature-induced hypocotyl elongation
