# Supplementary material for: Natural variants of ELF3 affect thermomorphogenesis by transcriptionally modulating PIF4-dependent auxin response genes
Source: BMC Plant Biol. 2015 Aug 14;15:197. doi: 10.1186/s12870-015-0566-6 (PMC4535396; doi:10.1186/s12870-015-0566-6)
Supplement: Additional file 8: — Methods S1. Estimation of heritability and QTL analysis procedure. [file 12870_2015_566_MOESM8_ESM.pdf]

Supplementary Methods S1

Estimation of the broad sense heritability in the RIL population

The broad sense heritability (H^2) for hypocotyl length and hypocotyl growth elongation for the RIL population was estimated according to the formula: $H^2 = \sigma^2_g/(\sigma^2_g + \sigma^2_e)$, where σ^2_g is the between genotype variance component and σ^2_e is the within residual (error) variance component. Using a random-effects model (model 2) one-way ANOVA according to the model $\gamma_{ij} = \mu + g_i + e_{ij}$, the total phenotypic variance for each trait was partitioned and the mean square of between (MSB) groups and the mean square of within (MSW) groups were obtained. With these estimates σ^2_g was then calculated as $\sigma^2_g = (MSB - MSW)/n$, where n is the number of replicates and σ^2_g was defined as $\sigma^2_e = MSW$.

For the trait hypocotyl length, each measured plant of each line in the RIL population was considered as a replicate to estimate the broad sense heritability. For the trait hypocotyl growth elongation, which is calculated as the ratio of mean values of several plants between a control and a treatment condition, three replicates were obtained by randomly choosing a subset of plants (5 out of 15) for the control and the treatment condition for three times and calculating the mean values for the sampled subsets. This subsampling was repeated 1000 times and average broad sense heritability was calculated.

QTL analysis

The defined phenotypic RIL mean values were used for the QTL mapping. Prior to the QTL analysis, the phenotypic data were checked for normal distribution and outliers were excluded from further analysis. The complete table of values used for the analysis is available as Supplemental Dataset 1. To detect main effect QTLs, interval mapping and composite interval mapping was used with the Haley-Knott regression. The conditional genotype probabilities were calculated using the 'calc.genoprob' function with a step size of 1 cM and an assumed genotyping error probability of 0.001 using the Kosambi map function as implemented in the R qtl package (Broman *et al.*, 2003). For composite interval mapping, the 'cim' function was used with a pre-defined number of covariates selected by a forward approach and a window size of 10 cM. For each analyzed trait a genome-wide logarithm of ods (LOD) score threshold was estimated by 1000 permutations to correct for type I error rates of alpha = 0.05 as suggested by Churchill & Doerge (1994). If a QTL LOD score crosses the LOD score threshold it was declared as significant and all detected QTLs were further used as initial QTL models for the 'stepwiseqtl' function. Prior to the 'stepwiseqtl' function for each trait individual "heavy" and "light" penalties were extracted from 1000 'scantwo' permutations with the 'calc.penalties' function as described by Manichaikul *et al.* (2009) (for a detailed description see Broman & Sen (2009) and the documentation of the 'stepwiseqtl' function). The multiple QTL models for each trait defined with the 'stepwiseqtl' function were finally used with the 'makeqtl' and 'fitqtl' function to explore the estimated QTL effects and % variance explained by a QTL. The LOD support interval for each QTL in the multiple QTL model was estimated as the 95% Bayes credible intervals with the 'bayesint' function and the lowest and highest values are indicated in Table S1.
